# Radioactive ^125^I seeds inhibit cell growth and epithelial-mesenchymal transition in human glioblastoma multiforme via a ROS-mediated signaling pathway

**DOI:** 10.1186/1471-2407-15-1

**Published:** 2015-02-19

**Authors:** Yunhong Tian, Qiang Xie, Jie He, Xiaojun Luo, Tao Zhou, Ying Liu, Zuoping Huang, Yunming Tian, Dan Sun, Kaitai Yao

**Affiliations:** Cancer Research Institute, Southern Medical University, Guangzhou 510, 515 Guangdong Province, People’s Republic of China; Department of Radiation Oncology, Cancer Center of Guangzhou Medical University, Guangzhou, Guangdong Province People’s Republic of China; Department of Oncology, Armed Police Corps Hospital of Guangdong Province, Guangzhou, People’s Republic of China

**Keywords:** Irradiation, Radioactive ^125^I seeds, Glioblastoma multiforme, Epithelial-mesenchymal transition

## Abstract

**Background:**

Glioblastoma multiforme (GBM) is the most common primary central nervous system neoplasm in adults. Radioactive ^125^I seed implantation has been widely applied in the treatment of cancers. Moreover, previous clinical trials have confirmed that ^125^I seeds treatment was an effective therapy in GBM. We sought to investigate the effect of ^125^I seed on GBM cell growth and Epithelial-mesenchymal transition (EMT).

**Methods:**

Cells were exposed to irradiation at different doses. Colony-formation assay, EdU assay, cell cycle analysis, and TUNEL assay were preformed to investigate the radiation sensitivity. The effects of ^125^I seeds irradiation on EMT were measured by transwell, Boyden and wound-healing assays. The levels of reactive oxygen species (ROS) were measured by DCF-DA assay. Moreover, the radiation sensitivity and EMT were investigated with or without pretreatment with glutathione. Additionally, nude mice with tumors were measured after treated with radiation.

**Results:**

Radioactive ^125^I seeds are more effective than X-ray irradiation in inhibiting GBM cell growth. Moreover, EMT was effectively inhibited by ^125^I seed irradiation. A mechanism study indicated that GBM cell growth and EMT inhibition were induced by ^125^I seeds with the involvement of a ROS-mediated signaling pathway.

**Conclusions:**

Radioactive ^125^I seeds exhibit novel anticancer activity via a ROS-mediated signaling pathway. These findings have clinical implications for the treatment of patients with GBM by ^125^I seeds.

## Background

Glioblastoma multiforme (GBM) is the most common and lethal type of primary central nervous system neoplasm in adults [1]. Unlike most other tumors that metastasize to distant organs, malignant glioma very rarely metastasizes outside the central nervous system. In this sense, GBM may be regarded as a “local” tumor [2]. In GBM, standard treatment involves maximal resection followed by concomitant and adjuvant chemoradiotherapy with temozolomide. Even with this comprehensive treatment strategy, outcomes for patients with this malignancy remain very poor. Thus, in order to improve the current therapeutic regimens, it is important to explore effective new modalities for GBM patients. Radioactive ^125^I seed implantation has been widely applied in the treatment of cancers [3–6]. It has been shown to be an effective adjuvant therapy in recurrent GBM [7, 8] and Low-grade (WHO grades I and II) gliomas (LGGs) [9, 10]. Furthermore, several studies have shown that ^125^I seed irradiation directly causes more cell death by comparing with ^60^CO-γ or X-ray irradiation [11–14]. However, few studies of the biological effects of ^125^I seed irradiation on GBM cells are available.

Epithelial–mesenchymal transition (EMT) is a key developmental program that is often activated during cancer invasion and metastasis [15]. Cells that have undergone EMT are resistant to many of the chemotherapeutic and adjuvant drugs that are used to treat epithelial tumors, and may therefore drive tumor recurrence [16, 17]. For the lack of E-cadherin expression in GBM cells suggesting a non-classic EMT, only very few recent reports described an EMT phenomenon in GBMs and its association with the poor prognostic mesenchymal subgroup of GBMs [18]. Reactive oxygen species (ROS) play an important role in cellular metabolism and cancer therapy [19, 20]. The absorption of ionizing radiation by living cells can act indirectly through radiolysis of water, thereby generating ROS [21]. Moreover, in the past few years, nuclear DNA damage-sensing mechanisms activated by ionizing radiation have been identified, including ataxia-telangiectasia mutated (ATM)/ATM-and Rad3-related (ATR) and the DNA-dependent protein kinase [22, 23].

Therefore, in this study, we evaluated the effect of radioactive ^125^I seeds on GBM cell growth and EMT. The results showed that radioactive ^125^I seeds were more effective than X-ray irradiation in inhibiting GBM cell growth. Moreover, EMT in GBM cells was effectively inhibited by ^125^I seed irradiation. A mechanism study indicated that GBM cell growth and EMT were inhibited by ^125^I seeds with the involvement of a ROS-mediated signaling pathway. Pretreatment of cells with glutathione (GSH) significantly blocked ^125^I seed irradiation-induced inhibition of cell migration and growth by recovering the expression levels of ROS. Meanwhile, the results of an *in vivo* study confirmed that ^125^I seed irradiation inhibits tumor growth and EMT via a ROS-mediated signaling pathway. Taken together, these results suggest that radioactive ^125^I seeds exhibit novel anticancer activity via a ROS-mediated signaling pathway. These findings have clinical implications for the treatment of patients with GBM by ^125^I seeds.

## Methods

### Cell culture and reagents

U251 and U87 human GBM cell lines were available at the Cancer Institute of Southern Medical University (Guangzhou, China) and were originally purchased from the American Type Culture Collection (ATCC). Cells were maintained in Dulbecco’s Modified of Eagle Medium (DMEM) supplemented with 10% fetal bovine serum (FBS) and antibiotics (100 IU/ml penicillin and 100 mg/ml streptomycin) at 37°C under a humidified atmosphere of 95% air and 5% CO_2_. To investigate the effect of ROS on migration, 5 mM GSH (Sigma-Aldrich, MO, USA) was added 2 hours before irradiation.

### Treatment of GBM cells with ^125^I seeds and X-ray irradiation

^125^I seeds were obtained from Beijing Atom and High Technique Industries Inc. (Beijing, China). The *in vitro* irradiation was carried out as previously described [13]. The absorbed doses were calculated as follows: 44, 92, 144, and 204 hours were required for doses of 2, 4, 6, and 8 Gy, respectively [14]. X-ray irradiation with a clinically calibrated irradiation field of 10 × 10 cm was performed at the Department of Radiotherapy, Armed Police Corps Hospital of Guangdong Province, using the Elekta precise treatment system (Stockholm, Sweden).

### Colony-formation and thiazolyl blue tetrazolium bromide (MTT) assay

According to a previous study, the plating efficiency (PE) of unirradiated controls was calculated using the following formula: number of colonies/number of seeded cells × 100%. U87 and U251 cells were exposed to radiation and then seeded using a cell-dilution assay. Surviving fractions (SFs) were calculated as following formula: SF = number of colonies/number of seeded cells × PE. The dose–survival curve was fitted based on the single-hit multi-target theory formula: SF =1 - (1 - e^D/D0^) ^N^; logN = D_q_/D_0_. Cell viability was determined by MTT assay as previously described [24].

### Annexin V-PI apoptosis and Caspase-3 activity assay

Cells in exponential growth were irradiated and harvested 24 hours after irradiation. Then cells were assessed according to the protocol of the Alexa Fluor® 488 annexin V/Dead Cell Apoptosis kit (Invitrogen, CA, USA). For caspase-3 activity, cells incubated 48 hours after irradiation at different doses were lysed with lysis buffer (100 μl per 2 × 10^6^ cells) for 15 minutes on ice following washing with D-Hank’s medium. Then cell extracts mixed with Ac-DEVD-pNA substrate were incubated at 37°C for 2 hours. The values measured by colorimetric measurement of p-nitroanilide product at 405 nm were normalized to untreated controls allowing determination of the fold change in caspase-3 activity.

### Cell cycle measured by flow cytometry

Cells in exponential growth were irradiated and harvested 24 hours after irradiation. Then they were washed with cold phosphate-buffered saline (PBS) and fixed overnight in cold 70% ethanol. Fixed cells washed with PBS were resuspended in 100 μl RNaseA (250 μg/ml), incubated for 30 minutes at 37°C. Then, 50 μg/ml PI was added and incubated at room temperature in the dark for 30 minutes followed by PI-detection with BD FACSCAria™ (BD Biosciences, CA, USA).

### Analysis of apoptosis by terminal deoxynucleotidyl transferase (TdT)-mediated dUTP-digoxigenin nick-end labeling (TUNEL) assay

We applied a TUNEL assay according to the manufacturer’s instructions (Beyotime Institute of Biotechnology, Jiangsu, China) to evaluate the apoptotic response in tumor cells. Briefly, cells cultured on chamber slides were fixed with 3.7% formaldehyde and permeabilized with 0.1% Triton X-100 in PBS. Then, the cells were incubated with TUNEL reaction mixture for 1 hour and cell nuclei were stained with 4′, 6-diamino-2-phenylindole (DAPI; Invitrogen). The cells were then washed with PBS and examined.

### Transwell and Boyden chamber assays

Cells (10^6^ cells/100 μl) in serum-free DMEM were added to the upper chamber and 500 μl of the DMEM with 10% FBS was added to the lower chamber with permeable supports (Corning, NY, USA). Then, cells on the upper surface which were incubated for 24 hours at 37°C were removed using a cotton-tipped applicator. Finally, cells on the lower surface of the filter were stained with crystal violet to calculate the average number of migrated cells [25].

### Wound-healing assay

Cells exposed to irradiation at a dose of 4 Gy were scraped with a conventional 10 μl micropipette tip across the monolayer. The distance between the wound edges was measured immediately and again 24 hours later. The total distance migrated by wounded U251 and U87 cells was evaluated using Adobe Photoshop and is expressed as a percentage of the initial wound distance.

### Immunofluorescence assay

Cells seeded on slides were fixed in 4% paraformaldehyde and permeabilized in 0.5% Triton X-100. Primary antibody (1:200; Santa Cruz Biotechnology, CA, USA) and Alexa Fluor 488-conguated secondary antibody (1:500; Invitrogen) were used to detect the location and expression of E-cadherin and vimentin. The cell nuclei were stained with DAPI. Finally, the images were recorded by fluorescence microscopy with a Nikon eclipse 80i microscope.

### Detection of ROS in intracellular

For intracellular ROS analysis, cells were loaded with 10 μM DCF-DA (Sigma-Aldrich), incubated at 37°C for 30 minutes, and immediately analyzed by microscope and flow cytometry (BD Biosciences).

### Western blotting analysis

Cells and tissues were lysed in RIPA buffer. Tumors were ground in liquid nitrogen and lysed. Protein concentration was determined using the BCA Kit (Beyotime Institute of Biotechnology). Proteins were mixed with loading buffer and heated at 70°C for 10 minutes on sodium dodecyl sulfate (SDS)-polyacrylamide gels at 30 μg per lane. The proteins were transferred to polyvinylidene fluoride (PVDF, Millipore, MA, USA) after electrophoresis. Membranes were blocked for 2 hours in 5% BSA and incubated overnight at 4°C with antibodies against γ-H2AX, ATM, ATR, Chk1, cell-cycle controller-2 (Cdc2), E-cadherin, vimentin, caspase-3, and caveolin-1 (Cav-1). The blots were then incubated with HRP-conjugated secondary antibody (1:1000; Santa Cruz Biotechnology). Finally, bands were visualized by enhanced chemiluminescence (Thermo Scientific Pierce, IL, USA).

### *In vivo*experiments

Female BALB/c nude mice (age 4–5 weeks) were purchased from the Model Animal Research Center of Nanjing University (Nanjing, China). This study was approved by the ethics committee of Southern Medical University. Animals were injected subcutaneously (s.c.) with U251 cells into the right hind limb (5 × 10^6^ cells/100 μl). Two weeks later, mice whose tumor volumes had reached approximately 200 mm^3^ were randomly divided into three groups with 4 mice in every group. The three groups were: (1) irradiation at 20 Gy (2 Gy/day × 10 F, 5 fractions/week for X-ray irradiation); (2) implanted with ^125^I seeds at a total dose of 20 Gy, the number of which calculated by the treatment planning system (TPS) (RT-RSI, Beijing Atom and High Technique Industries Inc., Beijing, China); and (3) untreated group. The dimensions of xenograft nodules were callipered every 3 days for 2 successive weeks. The animals were euthanized day 15 after treatment. Finally, immunohistochemistry (IHC) and western blotting for E-cadherin and vimentin were performed in xenograft tumor samples.

### Statistical analysis

Statistical analysis was performed with the SPSS statistical package (v15.0). *In vitro* experiments were usually performed in triplicate and repeated three times. The data are presented as mean ± standard deviation (SD). Statistical differences among groups were examined using one-way analysis of variance (ANOVA), with *p* values of less than 0.05 considered statistically significant. Multiple comparisons of the means were done by the least significance difference (LSD) test.

## Results

### Radioactive ^125^I seeds are more effective than X-ray in inhibiting GBM cell growth

**Figure 1:**
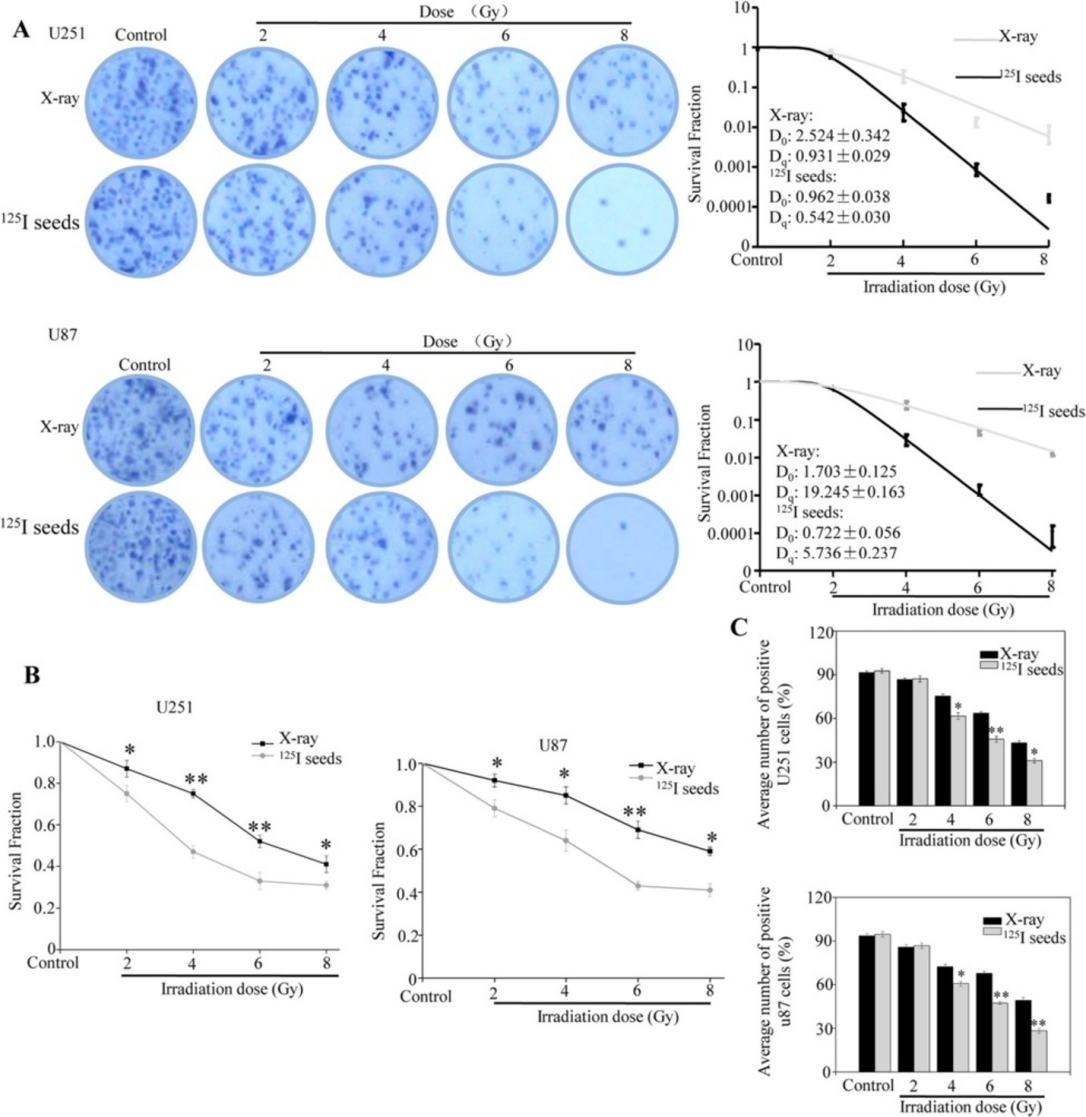
**Glioblastoma multiforme (GBM) cells are more radiosensitive to** ^**125**^ **I seed irradiation than X-ray. (A)** Representative pictures of the colony-formation ability of U251 (upper panel) and U87 (lower panel) cells exposed to ^125^I seed and X-ray at various doses for 14 days. The survival fraction of the colony-formation assay was fitted by the single-hit multi-target theory formula. **(B)** The cell viability of U251 (left panel) and U87 (right panel) cells treated with irradiation was examined by MTT assay. **(C)** Proliferation of U251 (upper panel) and U87 (lower panel) cells was measured by EdU assay. Significant differences between the ^125^I seed and X-ray groups under the same dose are indicated by *P <0.05 and **P < 0.01.

In this study, the effects of irradiation on the growth of GBM cells were measured by colony-formation, MTT, and apoptosis assay. The results showed that the colony-formation ability was significantly reduced by irradiation in a dose-dependent manner (Figure 1A). The SFs of cells exposed to ^125^I seed irradiation were significantly lower than that of cells exposed to X-ray irradiation at the same doses, both in U251 and U87 cells. Based on the dose-survival curve fitted with the single-hit multi-target theory formula, the D_0_ and D_q_ of ^125^I seeds and X-ray were 1.703 versus 0.722 and 19.245 versus 5.736 in U87. Moreover, the MTT assay also confirmed that the viability of cells exposed to ^125^I seeds was lower than that of cells exposed to X-ray irradiation (Figure 1B), especially at a dose of 4 Gy; the SF decreased from 77.63% to 47.57% in U251 cells and from 84.42% to 61.69% in U87 cells. Finally, cell proliferation was measured and the results indicated that the cell proliferation of U251 and U87 was significantly inhibited by irradiation in a dose-dependent manner. However, compared with the X-ray group, the cell proliferation was significantly inhibited by ^125^I seeds at the same doses (Figure 1C). Taken together, these assays indicate that GBM cells are more sensitive to ^125^I seed irradiation than to X-ray irradiation.

**Figure 2:**
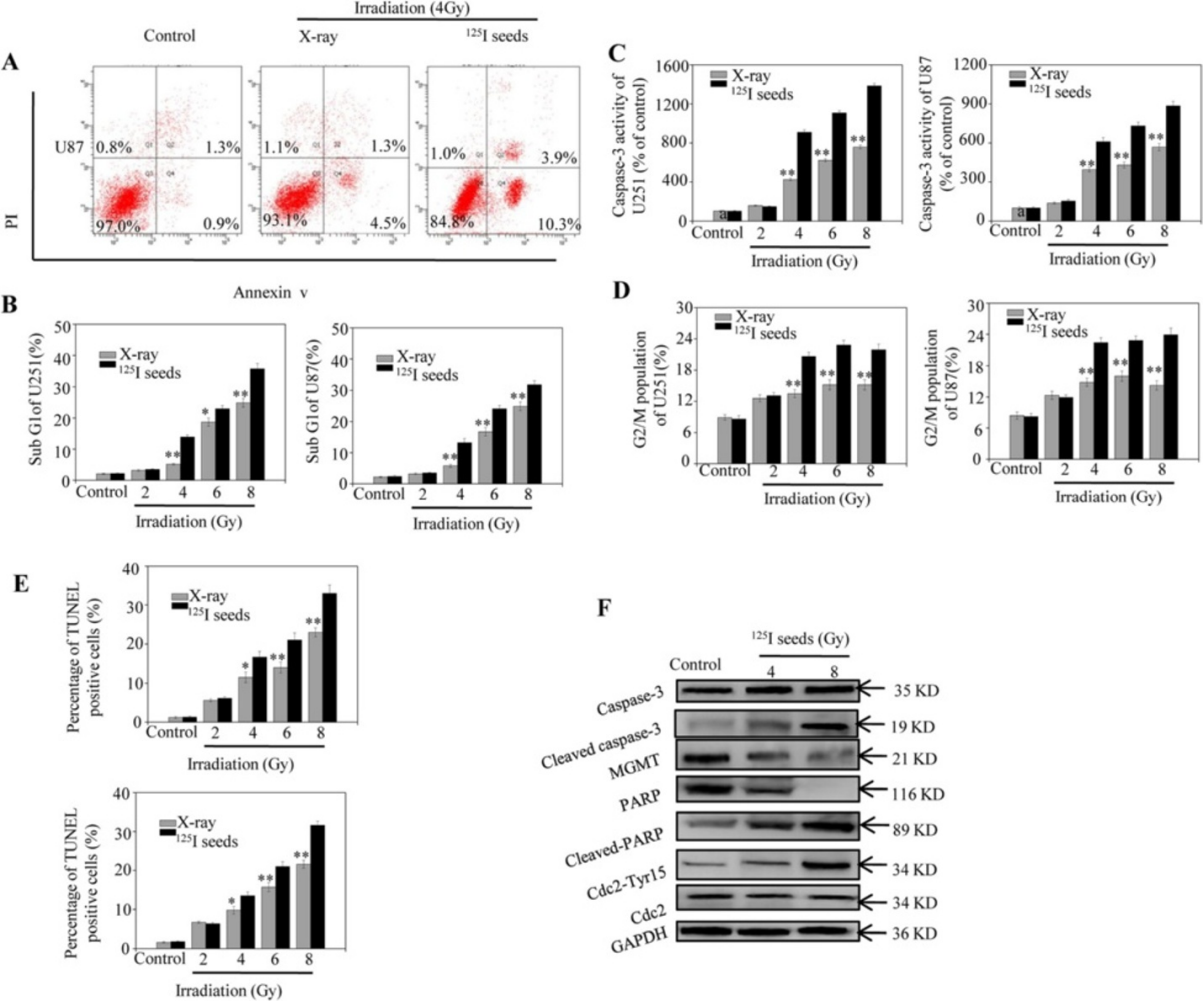
**Apoptosis and G2/M arrest in glioblastoma multiforme (GBM) cells is induced by** ^**125**^ **I seed irradiation.** Apoptosis of GBM cells was examined by Annexin V-PI co-staining flow cytometric analysis **(A, B)** and caspase-3 activity assay **(C)**. **(D)** Effects of ^125^I seed on the cell-cycle distribution of GBM cells were examined by flow cytometric analysis. **(E)** TUNEL assay was used to evaluate the apoptotic response in U251 (left panel) and U87 (right panel) cells. **(F)** Treatment of cells with ^125^I seeds caused obvious apoptosis in a dose-dependent manner, accompanied by up-regulation of cleaved caspase-3, cleaved PARP, and phosphorylated Cdc2 (Tyr 15). Data are presented as mean ± SD (n =3). Significant differences between the ^125^I seed and X-ray groups under the same dose are indicated by *P <0.05 and **P < 0.01.

An annexin V– PI apoptosis assay was performed to examine the effect of ^125^I seed irradiation on cell death. The results showed that apoptotic cell death was markedly induced by X-ray and ^125^I seed irradiation in a dose-dependent manner. After X-ray irradiation, apoptosis significantly increased in GBM cells. However, compared with X-ray irradiation, ^125^I seed irradiation led to a higher percentage of apoptosis under the same doses. For example, the apoptotic rate after ^125^I seed irradiation at a dose of 4 Gy increased from 2.12% (control) to 15.21% in U251 cells and to 14.26% in U87 cells (Figure 2A and B). We further investigated whether the observed irradiation-induced apoptosis was related to caspase-3. As expected, the results showed that caspase-3 activity increased after irradiation, with ^125^I seed irradiation exhibiting a greater effect on caspase-3 activity compared with X-ray (Figure 2C). To gain a better understanding of the apoptosis induced by the ionizing irradiation, DNA distribution histograms of GBM cells were acquired. The results indicated that dose-dependent increases in the G2/M cell population were observed in cells exposed to X-ray and ^125^I seed irradiation, without significant differences in the S and G0/G1 phases (Figure 2D). As we know, TUNEL-positive cells showed typical apoptosis. The results of our study indicate that the number of TUNEL-positive cells was significantly increased with ^125^I seed irradiation as compared with X-ray irradiation at the same doses (Figure 2E). With irradiation at a dose of 4 Gy, the percentage of apoptotic cells increased to 19.18% with ^125^I seed treatment, compared with 12.32% with X-ray. The enzyme poly-ADP-ribosepolymerase (PARP), the expression of which is triggered by DNA-strand breaks, acts as a substrate for caspases. In cells undergoing apoptosis, it is cleaved by caspase-3 during the degradation of cellular DNA, thus preventing DNA damage repair. It is therefore critical for the repair of some DNA lesions [26, 27]. Historically, mitotic entry and exit were thought to be a direct consequence of Cdc2 activation and inactivation, respectively [28]. Therefore, PARP, caspase-3, and Cdc2 in U251 were measured by western blotting to further confirm the apoptosis and G2/M arrest induced by ^125^I seeds. As expected, treatment of cells with ^125^I seeds caused obvious apoptosis and G2/M arrest in a dose-dependent manner, as accompanied by up-regulation of cleaved caspase-3, cleaved PARP, and phosphorylated Cdc2 (Tyr 15). Interestingly, results indicated that O6-methylguanine DNA methyltransferase (MGMT) which was an independent favorable prognostic factor in patients with GBM [29] was also up-regulated by irradiation (Figure 2F). Altogether, these results suggest a higher potency of ^125^I seed irradiation in inducing cancer-cell apoptosis and G2/M arrest.

### Effects of irradiation on EMT in GBM cells

**Figure 3:**
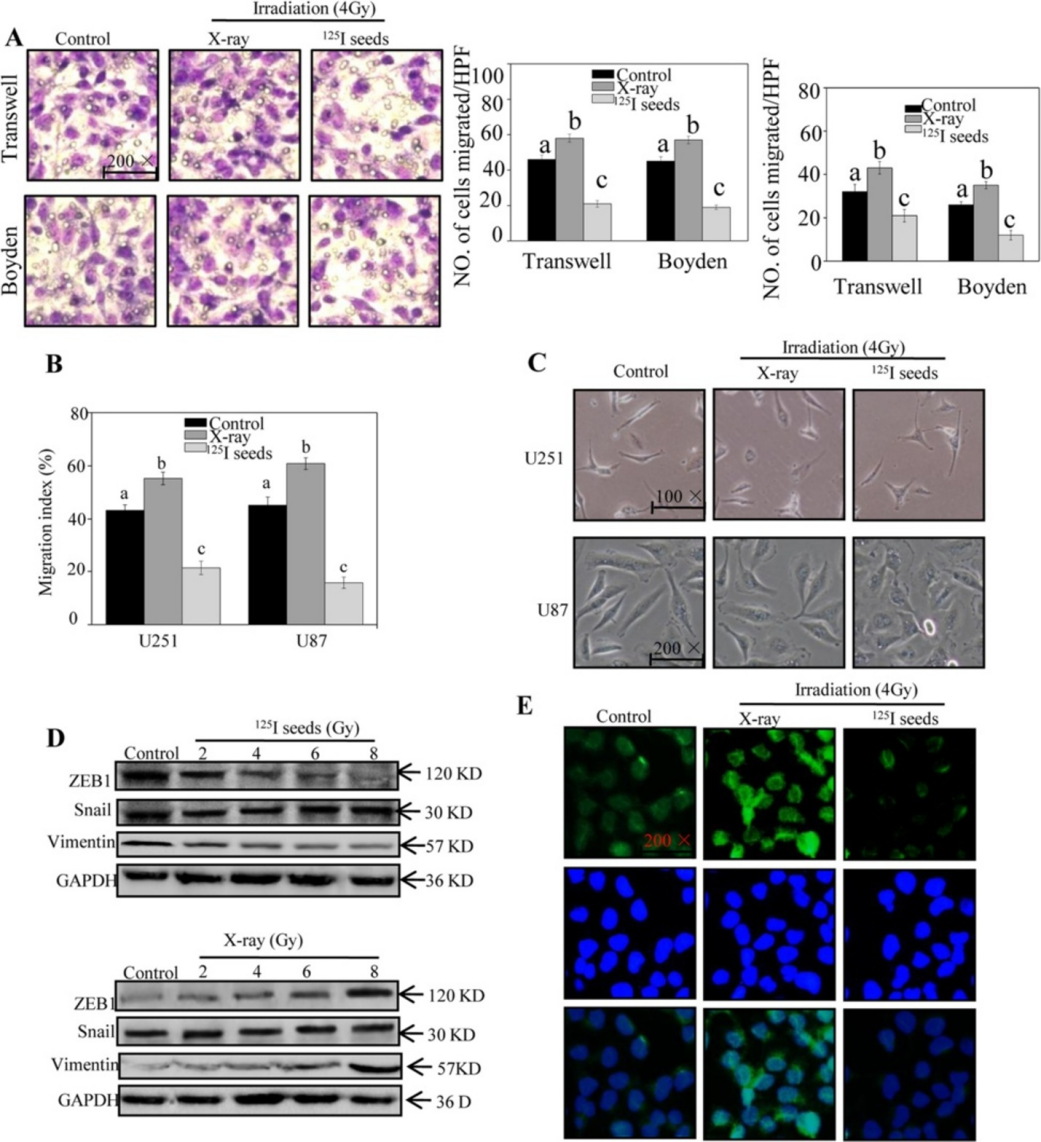
**Effects of** ^**125**^ **I seed irradiation on epithelial–mesenchymal transition (EMT) in glioblastoma multiforme (GBM) cells. (A)** The inhibition of cell invasion was measured by transwell and Boyden chamber assays. The number of U251 (left panel) and U87 (right panel) cells was counted to calculate the average number of migrated cells. **(B)** A wound-healing assay of GBM cells was performed followed by irradiation at a dose of 4 Gy, and the U251 cells were presented. The total distance migrated by wounded cells is expressed as a percentage of the initial distance. **(C)** Morphological changes of U251 (upper panel) and U87 (lower panel) cells treated by irradiation. **(D)** The expressions of ZEB1, Snail, and vimentin in U251 cells treated with ^125^I seeds (upper panel) and X-ray (lower panel) were measured by western blotting. **(E)** ZEB1 of U251 cells were measured by immunofluorescent assay. Data are presented as mean ± SD (n =3). Bars with different characters are statistically different at the *P* < 0.05 level.

GBM cells are characterized by migration and invasion into the surrounding normal brain tissue. This study compared the EMT in GBM cells with X-ray and ^125^I seed irradiation. As shown in Figure 3A, transwell and Boyden chamber assays were performed to investigate the effects of these treatments on cell invasion and migration. After X-ray irradiation, the number of migrating cells per high-power field (HPF) in U251 cells increased from 42.3 to 59.4, whereas it reduced to 22.9 after ^125^I seed irradiation. Similar findings were seen with U87 cells: the number of migrating U87 cells decreased significantly after ^125^I seed irradiation. When the potential involvement of ^125^I seeds in reducing the invasion of GBM cells was determined by Boyden chamber assay, there was a two-fold decrease in the number of invading cells of both U251 and U87 compared with the control group. As shown in Figure 3B, the migration index of ^125^I seed irradiation measured by wound-healing assay reduced from 40.9% to 22.1% compared with control. However, a higher migration index of U251 cells was observed 24 hours after X-ray irradiation. Moreover, dramatic morphological changes, in which the spindle-like, fibroblastic morphology was replaced by a typical cobblestone-like appearance of normal epithelium, indicating inhibition of EMT by ^125^I seeds, were observed in ^125^I seed-treated GBM cells (Figure 3C). In GBM cell lines, the capabilities of motility and invasion have been ascribed to Snail and ZEB1 expression [30]. Thus, our studies investigated the levels of ZEB1 and Snail. Our results indicated that the levels of ZEB1 were involved in EMT inhibited by ^125^I seeds, with no significant changes in Snail in U251 and U87. As anticipated, decreased vimentin were also observed in ^125^I seed-treated U87 and U251 cells (Figure 3D). To further test the hypothesis that ^125^I seeds inhibit EMT, the results of immunofluorescence assay indicated that ^125^I seeds down-regulated ZEB1 in the nuclear of U251 cells, suggesting that ^125^I seeds inhibit EMT in GBM cells (Figure 3E). Taken together, these results demonstrate that ^125^I seed irradiation inhibits the nonclassic EMT in GBM cells.

### Radioactive ^125^I seeds suppress cell migration and growth via a ROS-mediated signaling pathway

**Figure 4:**
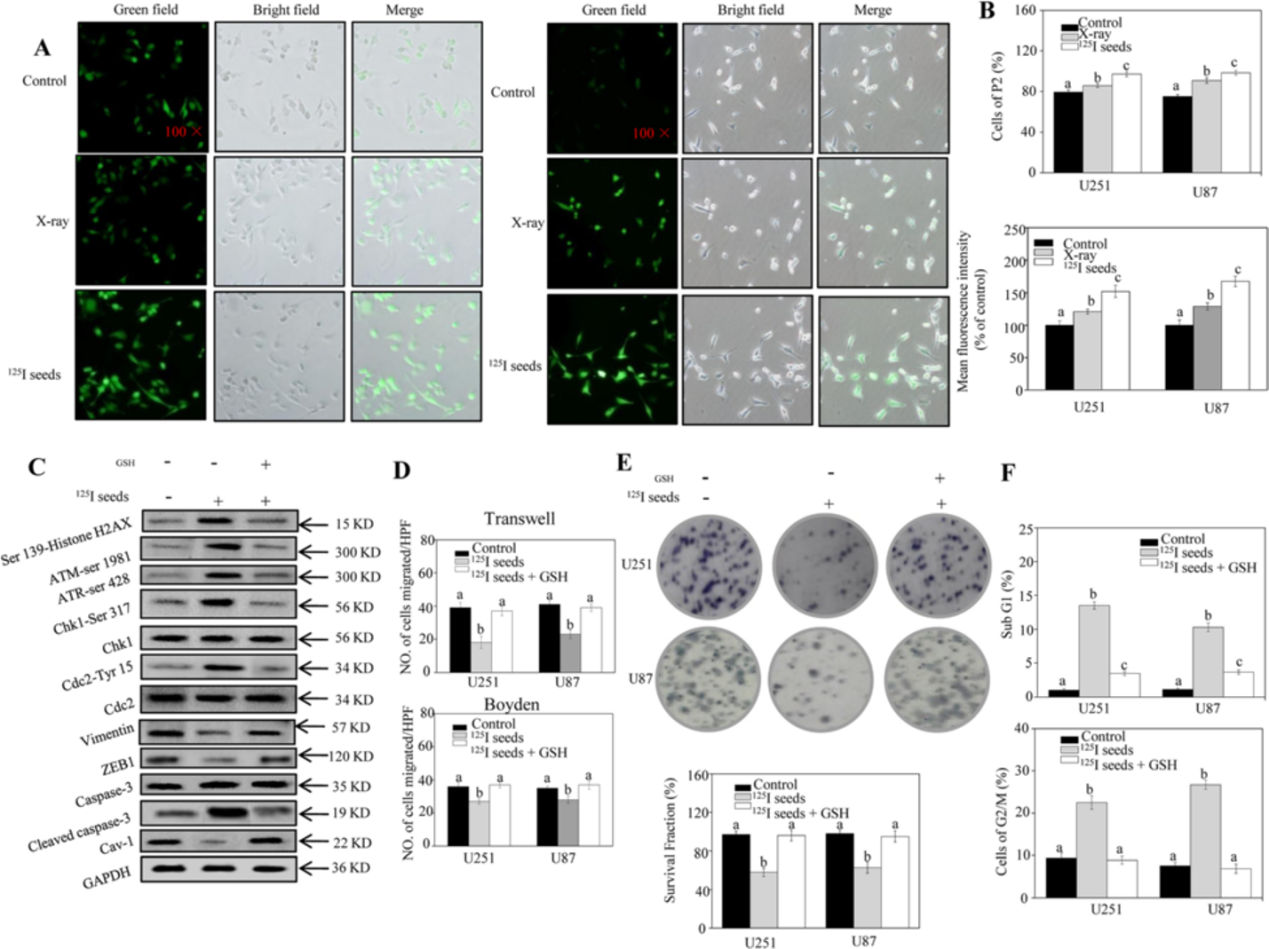
**Radioactive** ^**125**^ **I seeds suppress epithelial–mesenchymal transition (EMT) in glioblastoma multiforme (GBM) cells via a reactive oxygen species (ROS)-mediated signaling pathway. (A)** The levels of ROS in U251 (left panel) and U87 (right panel) cells were measured by DCF-DA staining 24 hours after ^125^I seed and X-ray treatment. The green fluorescence intensity indicates the degree of ROS. **(B)** The levels of ROS in ^125^I seed- and X-ray-treated GBM cells were measured by flow cytometric analysis. The mean fluorescence intensity and the cells of P2 measured by flow cytometric analysis are presented in. **(C)** Pretreatment of cells with GSH obviously decreased DNA damage and apoptosis in U251 cells, reflected by γH2AX, activated ATM, ATR, Chk1, and Cdc2. EMT was also recovered by GSH. **(D)** The number of cells measured by transwell (upper panel) and Boyden (lower panel) assay was counted to calculate the average number of migrated cells when GSH was used. Moreover, colony-formation ability of GBM cells **(E)** of U251 cells, and DNA distribution **(F)** of U251 cells inhibited by ^125^I seeds were blocked by recovery of the expression levels of ROS. Data are presented as mean ± SD (n =3). Bars with different characters are statistically different at the *P* < 0.05 level.

Studies have shown that ROS play an important role in cancer therapy; in particular, low- linear energy transfer (LET) radiation exposure is more dependent on ROS generation. Therefore, we hypothesized that ^125^I seeds may inhibit cell growth and EMT via increased ROS generation in GBM cells. To test this hypothesis, we sought to determine whether ^125^I seeds were more effective than X-ray in generating ROS. DCF-DA staining and flow cytometric assays showed that the levels of ROS were markedly increased in U251 and U87 cells after 24 hours of ^125^I seed irradiation. As shown in Figure 4A, the degree of ROS was measured under a microscope, with green fluorescence indicating the creation of ROS. Results indicated that ^125^I seeds stimulated higher level of ROS in both U87 and U251 cells, as reflected by the intensity of green fluorescence and the percentage of cells carrying ROS. In addition, flow cytometry analysis revealed that ^125^I seeds were more effective in stimulating ROS generation than X-ray irradiation at the same doses (Figure 4B). For example, the percentage of U251 cells carrying ROS increased from 79.3% to 97.1%. Moreover, the mean fluorescence intensity measured by flow cytometry significantly increased by 1.5-fold in U251 cells and 1.8-fold in U87 cells, compared with the control group. Taken together, these results suggest that the inhibition of GBM cell growth and EMT induced by ^125^I seeds may be associated with ROS generation.

Increased ROS is also known to produce DNA double-strand breaks (DSBs) with accumulation of the known marker γH2AX. ATM and its downstream kinase Chk1 phosphorylate several targets that regulate DNA repair, cell-cycle checkpoints, and apoptosis [31, 32]. Other studies have implicated ROS in the aggressive behavior of cancer [33]. Moreover, we have found that ^125^I seeds can induce ROS production. Therefore, we hypothesized that ^125^I seeds may lead to inhibition of cell growth and invasion via a ROS-mediated signaling pathway. To test this hypothesis, western blotting was performed and GSH, which can scavenge ROS, was used. The results indicated that pretreatment of cells with GSH obviously decreased DNA damage, G2/M arrest, and apoptosis (Figure 4C). Interestingly, ^125^I seed-inhibited EMT associated proteinrecovered when GSH was applied. To further test whether EMT and cell growth were inhibited via a ROS-mediated signaling pathway, Invasion and growth of GBM cells were measured with and without GSH. The results indicated that pretreatment of cells with GSH recovered the cell invasion suppressed by ^125^I seeds (Figure 4D). The inhibited colony-formation in GBM cells was also rescued by GSH (Figure 4E). Moreover, when cells were pretreated with GSH, ^125^I seed-induced cell apoptosis, as reflected by sub-G1, decreased from 13.46% to 1.72%, and the percentage of G2/M cells decreased from 22.85% to 8.23% in U251 (Figure 4F). Taken together, these results suggest that radioactive ^125^I seeds suppress cell migration and cell growth by activating a ROS-mediated signaling pathway, and that GSH blocks the ^125^I seed irradiation-induced inhibition of cell migration and cell growth.

### Radioactive ^125^I seeds exhibit greater *in vivo*anticancer activity than X-ray

**Figure 5:**
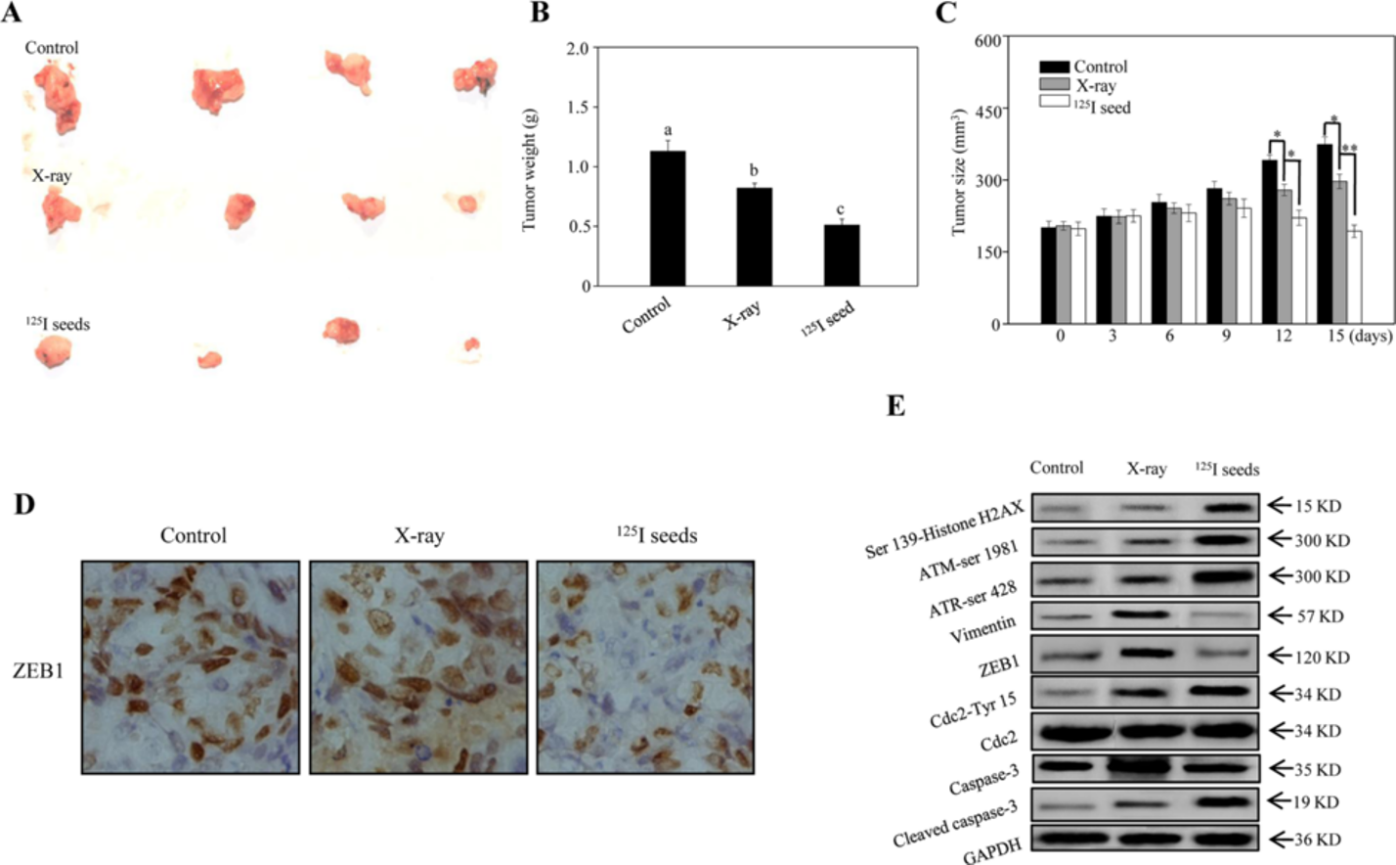
^**125**^ **I seed irradiation is associated with greater** ***in vivo*** **anticancer activity than X-ray. (A)** Representative images of tumors treated with ^125^I seed and X-ray irradiation. **(B, C)** tumor weight and tumor size were measured after irradiation. Data are presented as mean ± SD (n =3). Significant differences between the ^125^I seed and X-ray groups under the same dose are indicated by *P < 0.05 and **P < 0.01. **(D, E)** Proteins with the involvement of cell growth and epithelial–mesenchymal transition were further detected by IHC and western blotting.

An *in vivo* experiment was performed to further evaluate the effect of ^125^I seed irradiation. This study was performed according to TPS to ensure consistency with clinical therapy. As anticipated, both X-ray and ^125^I seed irradiation at a cumulative dose of 20 Gy significantly inhibited xenograft tumor growth *in vivo* (Figure 5A and B). Measurement of xenograft tumor volumes showed that the sizes of tumors deriving from the ^125^I seed and X-ray groups were significantly smaller than those from the control group by day 12 (Figure 5C). Interestingly, compared with X-ray irradiation, ^125^I seed irradiation more effectively inhibits tumor growth. Moreover, the expression of proteins associated with DNA damage, G2/M, apoptosis and EMT were detected in xenograft tumors by western blotting and IHC. The *in vivo* results indicate that ^125^I seeds caused up-regulation of γH2AX and of the phosphorylation levels of ATM (Ser 1981), ATR (Ser 428), and Cdc2 (Tyr 15), indicating that ^125^I seeds are more effective than X-ray in inhibiting cell growth. Moreover, EMT was inhibited by ^125^I seeds, as reflected by decreased vimentin and ZEB1 (Figure 5D and E). Interestingly, the body weight of the nude mice exposed to X-ray irradiation decreased more significantly than that of the ^125^I irradiation group (data not shown). Taken together, these findings indicate that exposure to ^125^I seed radiation is more effective than X-ray in inhibiting GBM cell growth and EMT *in vivo*.

## Discussion and conclusions

GBM is the most common primary central nervous system neoplasm in adults. The annual incidence of malignant gliomas in Western countries is approximately 5 cases per 100,000 people [1]. Unlike most other tumors, extracranial metastases from GBM are extremely rare [2]. In GBM, standard treatment involves maximal resection followed by concomitant and adjuvant chemoradiotherapy with temozolomide. Even with complete resection, tumors will recur because of the infiltrative and diffuse nature of GBM and its intrinsic radioresistance [34]. Although the comprehensive treatment strategy for GBM has continuously progressed over recent decades, patients with GBM still have an extremely poor prognosis with median survival of 14.6 months [35]. Thus, the discovery of novel treatments that can overcome the diffuse nature of GBM and its intrinsic radioresistance has been an aspiration of scientists in the fields of oncology. Interestingly, previous clinical trials have indicated that ^125^I seeds treatment is an adjuvant therapy to be effective in recurrent GBM [8, 36]. Stereotactic brachytherapy (SBT) with ^125^I seeds was confirmed to be an effective and safe treatment for patients with small unresectable or incompletely resected LGG. Moreover, it has been confirmed that radioactive ^125^I seed inhibited the cell growth, migration, and invasion of NPC cells [12]. These encouraging results compel us to embark on testing larger numbers of cancer with this treatment modality. Thus, the current study was performed to investigate the biological effects of ^125^I seeds on GBM. Results confirmed that radioactive ^125^I seeds are more effective than X-ray irradiation in inhibiting GBM cell growth. Moreover, EMT in GBM cells was effectively inhibited by ^125^I seed irradiation *in vitro* and *in vivo*. Further studies indicated that the anticancer effect of ^125^I seed irradiation was achieved via a ROS-mediated signaling pathway.

Previous studies have reported that ^125^I seed irradiation is more effective than ^60^CO-γ ray irradiation in inhibiting cell growth in PANC-1, PC3 prostate cancer, and CL187 colonic cells [12, 14]. Therefore, we evaluated the radiosensitivity of GBM cells. The results showed that GBM cells are more sensitive to ^125^I seed irradiation than to X-ray irradiation. Moreover, a significant increase in the proportion of apoptosis with the involvement caspase-3 and G2/M arrest was observed after ^125^I seeds irradiation. Similarly to our results, a prolonged accumulation in the G2/M phase and inhibited growth after exposure to ^125^I seed irradiation has been previously described [12, 14]. EMT is a tightly regulated process that occurs during tumor cell metastasis. Without knowledge of the underlying mechanisms, a previous study reported that a sublethal dose of X-ray irradiation promoted the migration and invasion of glioma cells in the border area during postoperative radiotherapy [37]. In contrast, some studies have shown that both proton and carbon ion irradiation significantly decreased cell migration and invasion [38, 39]. Therefore, in our study, the effects of ^125^I seed and X-ray irradiation on GBM cell migration were measured. Interestingly, we showed that X-ray irradiation promoted the migration and invasion of GBM cells, while inhibitory effects were observed in the ^125^I seed irradiation group. Consistently, EMT of NPC cell lines were inhibited by ^125^I seeds [12]. Currently, Kim et al. found that activation of the Snail pathway by X-ray was important for radioresistance primarily via EMT induction [40]. Mahabir et al. also described that GBM post irradiation undergo EMT and stemness features throughout the early to late phases by upregulating Snail [30]. Gomez et al. confirmed that non-small cell lung cancer cells survived X-ray irradiation treatment display cancer stem cell and EMT phenotypes, with enhancement of motility and invasiveness and elevated resistance to apoptosis [41]. On the basis of these findings that EMT which can be induced by X-ray irradiation contributes to radioresistance, though a classic EMT is not involved in GBMs progression, we indicate that ^125^I seeds might be an effective treatment for GBM.

What could be the mechanisms behind the inhibition of GBM cell growth and invasion induced by ^125^I seed irradiation? Previous studies confirmed that ^125^I seed irradiation inhibited NPC cell line migration by inactivating VEGF-A/ERK signaling [11]. Kahlert et al. indicated that the activity of the canonical WNT/β-catenin signaling pathway was directly associated with increased motility of the GBM cells [18]. However, decreased WNT signaling or VEGF-A were not observed in GBM cells treated with ^125^I seeds (data not shown). As we all know, the absorption of ionizing radiation by living cells can act indirectly through radiolysis of water, thereby generating ROS that can damage nucleic acids, proteins, and lipids [21]. ROS play an important role in cancer therapy, particularly with low-LET irradiation [19, 42]. Therefore, we hypothesized that cell growth and invasion inhibition were induced by ^125^I seeds with the involvement of increasing ROS. The results indicated that ^125^I seed irradiation led to a higher level of ROS than X-ray under the same doses. Moreover, pretreatment of cells with GSH rescued the EMT and cell growth suppressed by ^125^I seeds. Taken together, these results indicate that radioactive ^125^I seeds inhibit GBM cell invasion and growth via a ROS signaling pathway, and that ROS inhibition can block the ^125^I seed irradiation-induced inhibition of cell migration and cell growth. Following this, the anticancer action of ^125^I seed and X-ray irradiation *in vivo* was investigated. As with the *in vitro* findings, the results indicate that ^125^I seeds exhibit greater anticancer activity than X-ray irradiation *in vivo*.

Increasing evidence suggests that up-regulated ROS induced by chemotherapy or radiotherapy is associated with increased EMT of cancer cells. In our study, however, the results indicate that ROS generated by ^125^I seeds can prevent GBM cells migration. This may be due to the type of ROS, and the dose of irradiation. Luanpitpong et al. have confirmed that superoxide anions and hydrogen peroxide down-regulate Cav-1 expression and inhibit cell migration and invasion, whereas hydroxyl radicals up-regulate Cav-1 expression and promote cell migration and invasion [33]. Interestingly, Cav-1 was confirmed to be down-regulated by ^125^I seeds in our study, and the inhibited EMT could be reversed by scavenger of ROS by GSH. Moreover, Urbich et al. have indicated that CD40 ligand inhibits endothelial cell migration by increasing the production of endothelial ROS, and that H_2_O_2_-prevented endothelial cell migration can be reversed vitamin C [43]. Although further studies need to perform to confirm the type of ROS generated by ^125^I seeds, we can conclude that ^125^I seeds can inhibit EMT and growth GBM cells through increased ROS. Effective treatment options are limited for patients with GBM, especially for recurrent GBM, thus, novel treatment approaches are needed. ^125^I seeds treatment is an adjuvant therapy that has been shown to be effective in recurrent GBM [7, 44]. Permanent placement of ^125^I seeds for recurrent GBM may prolong survival in patients with recurrent GBM [36]. Darakchiev et al. have indicated that the use of adjuvant therapy combining carmustine wafers and permanent ^125^I seeds was a good treatment option for patients with recurrent GBM who have undergone previous surgery and radiation therapy [8]. Overall, previous clinical trials have confirmed that ^125^I was an effective treatment for patients with GBM. However, our study was performed to investigate the biological effects of ^125^I seeds on GBM. Our results indicated that ^125^I seed irradiation was more effective than X-ray irradiation in inhibiting GBM cells via the ROS pathway. Obviously, our data are in line with the majority of published clinical trials studies. These results reported here also confirm and extend previous findings. Though GBM cell growth and invasion were inhibited by radioactive ^125^I seeds in this study, however the signaling pathway via which radioactive ^125^I seeds inhibited GBM cell growth and EMT in human GBM was ROS, which was different from previous study [11].

**Scheme 1:**
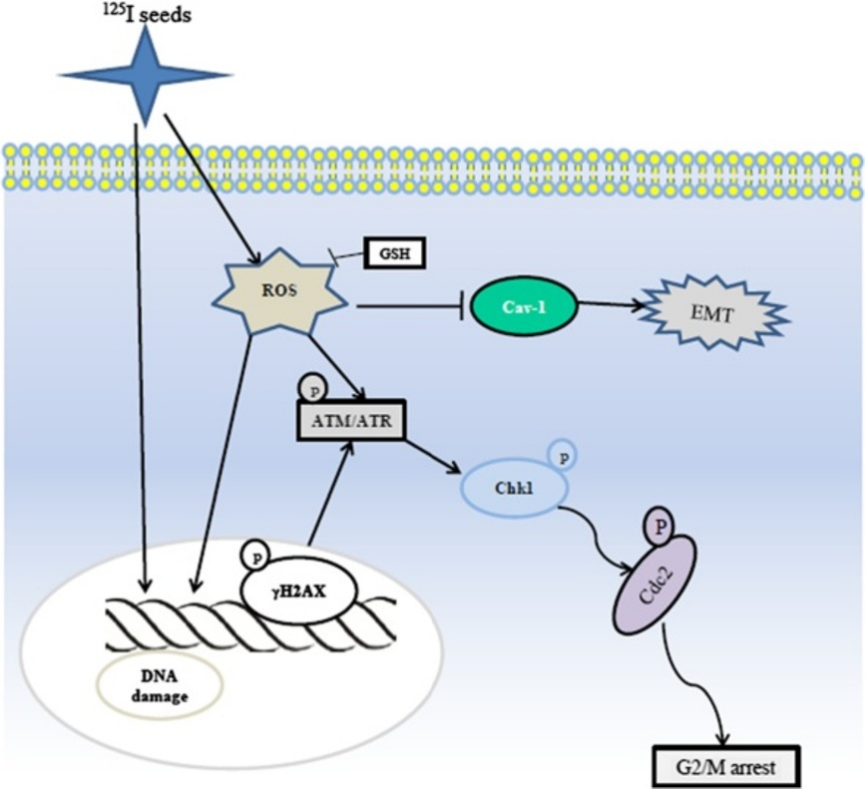
Proposed signaling pathway by which radioactive ^125^I seeds inhibit epithelial–mesenchymal transition (EMT) and cell growth in glioblastoma multiforme (GBM) cells **Proposed signaling pathway by which radioactive** ^**125**^ **I seeds inhibit epithelial–mesenchymal transition (EMT) and cell growth in glioblastoma multiforme (GBM) cells.** ^125^I seeds act indirectly through radiolysis of water, thereby generating ROS and producing DNA damage. DNA damage activates the sensory ATM/ATR kinases and finally results in cell growth inhibition and G2/M arrest. Moreover, ROS induced by ^125^I seeds can inhibit EMT via Cav-1 in GBM cells.

In summary, we have demonstrated, for the first time, that radioactive ^125^I seeds are more effective than X-ray irradiation in inhibiting GBM cell growth. Moreover, EMT of GBM cells was effectively inhibited by ^125^I seed irradiation. A mechanism study indicated that the cell growth and EMT inhibition in GBM cells was induced by ^125^I seeds with the involvement of a ROS signaling pathway. To our knowledge, this is the first attempt to study the effect of ^125^I seeds on GBM cells. These results suggest that radioactive ^125^I seeds exhibit novel anticancer activity via a ROS signaling pathway (Scheme 1). Furthermore, EMT of GBMs was inhibited by ^125^I seeds via ROS pathway. These findings have clinical implications for the treatment of GBM patients with ^125^I seeds. Based on the current report, ^125^I seeds either alone or in combination with other treatment may be a better choice for GBM patients. Future studies are needed to determine whether the biological effects of ^125^I seed irradiation identified in GBM cells can be reproduced in other cancer-cell types, thus broadening the significance of the data reported here.

## Abbreviations

Cav-1: Caveolin-1
Cdc2: Cell-cycle controller-2
CT: Computed tomography
DAPI: 4′ 6-diamino-2-phenylindole
EdU: 5-ethynyl-2′-deoxyuridine
EMT: Epithelial-mesenchymal transition
FBS: Fetal bovine serum
GBM: Glioblastoma multiforme
GSH: Glutathione
MTT: Thiazolyl blue tetrazolium bromide
PARP: Poly-ADP-ribose polymerase
PBS: Phosphate buffered saline
ROS: Reactive oxygen species
SD: Standard deviation
SF: Surviving fraction
TPS: Treatment planning system
TUNEL: Deoxynucleotidyl transferase (TdT)-mediated dUTP- digoxigenin nick-end labeling.
